# Piloting a Survey-Based Assessment of Transparency and Trustworthiness with Three Medical AI Tools

**DOI:** 10.3390/healthcare10101923

**Published:** 2022-09-30

**Authors:** Jana Fehr, Giovanna Jaramillo-Gutierrez, Luis Oala, Matthias I. Gröschel, Manuel Bierwirth, Pradeep Balachandran, Alixandro Werneck-Leite, Christoph Lippert

**Affiliations:** 1 Digital Engineering Faculty, University of Potsdam, 14482 Potsdam, Germany; 2 Digital Health & Machine Learning, Hasso Plattner Institute, 14482 Potsdam, Germany; 3 Milan and Associates SRL, 6960 Manhay, Belgium; 4 ITU/WHO Focus Group AI4H, 1211 Geneva, Switzerland; 5 Department of Artificial Intelligence, Fraunhofer HHI, 10587 Berlin, Germany; 6 Department of Biomedical Informatics, Harvard Medical School, Boston, MA 02115, USA; 7 Alumnus Goethe Frankfurt University, 60323 Frankfurt am Main, Germany; 8 Technical Consultant (Digital Health), Thiruvananthapuram 695010, India

**Keywords:** artificial intelligence for health, quality assessment, transparency, trustworthiness

## Abstract

Artificial intelligence (AI) offers the potential to support healthcare delivery, but poorly trained or validated algorithms bear risks of harm. Ethical guidelines stated transparency about model development and validation as a requirement for trustworthy AI. Abundant guidance exists to provide transparency through reporting, but poorly reported medical AI tools are common. To close this transparency gap, we developed and piloted a framework to quantify the transparency of medical AI tools with three use cases. Our framework comprises a survey to report on the intended use, training and validation data and processes, ethical considerations, and deployment recommendations. The transparency of each response was scored with either 0, 0.5, or 1 to reflect if the requested information was not, partially, or fully provided. Additionally, we assessed on an analogous three-point scale if the provided responses fulfilled the transparency requirement for a set of trustworthiness criteria from ethical guidelines. The degree of transparency and trustworthiness was calculated on a scale from 0% to 100%. Our assessment of three medical AI use cases pin-pointed reporting gaps and resulted in transparency scores of 67% for two use cases and one with 59%. We report anecdotal evidence that business constraints and limited information from external datasets were major obstacles to providing transparency for the three use cases. The observed transparency gaps also lowered the degree of trustworthiness, indicating compliance gaps with ethical guidelines. All three pilot use cases faced challenges to provide transparency about medical AI tools, but more studies are needed to investigate those in the wider medical AI sector. Applying this framework for an external assessment of transparency may be infeasible if business constraints prevent the disclosure of information. New strategies may be necessary to enable audits of medical AI tools while preserving business secrets.

## 1. Introduction

Artificial intelligence (AI) and machine learning applications offer the potential to transform healthcare systems by assisting healthcare providers in diagnostic decision-making [1,2]. Various AI-based prediction models for medicine have been developed [3,4,5,6], but limited generalizability to new application settings (medical, demographic, or location) through training with biased data often prevents their deployment in medical practice to avoid unintended harm to patients [7,8,9,10]. Transparency was recently defined by the WHO as a key ethical principle and requires information to be published before deployment to facilitate a meaningful public debate about the use of AI technology in healthcare [11]. The High-Level Expert Group of Artificial Intelligence (AI-HLEG), set up by the European Commission, issued guidelines for trustworthy AI and defined transparency as one dimension of trustworthiness that requires documentation on the intended use, used data, AI model, and application constraints [12].

Abundant guidance exists for providing transparency on clinical prediction models. These include guidance to report on the intended use; used datasets; development and validation steps [13,14,15,16,17,18]; clinical validations of AI models [19]; and clinical trials using AI interventions [20,21], templates, and checklists to provide transparency on AI tools to clinicians [22,23,24]. However, despite the existing guidance for transparent reporting, poorly reported medical AI models are still common [25,26,27], and the transparency required to achieve trustworthy AI, according to the AI-HLEG and WHO, remains unfulfilled. Two previous works provided internal assessment frameworks for organizations who want to assess if their AI tool meets the ethical expectations for trustworthy AI [28,29]. Other works have developed external assessment frameworks to uncover the technical and ethical issues among AI systems qualitatively from outside of the organization [30,31]. However, these frameworks do not explicitly assess if the transparency requirement for trustworthy AI is fulfilled. Our work intends to close this transparency gap for medical AI and provides a framework to assess the degree of transparency among medical AI tools.

Our framework includes a survey to prompt structured reporting about the intended use, AI model development and validation, ethical considerations, and caveats for deployment based on the existing guidelines for the transparent reporting of prediction models. After obtaining the reports, we assessed the degree of transparency by rating each survey response with either 0, 0.5, or 1, indicating if the required information was not, partially, or fully disclosed. Additionally, we assessed if the provided reports met the transparency requirements for trustworthy AI according to the ethical AI guidelines [11,12]. For this, we defined a set of trustworthiness criteria and scored responses to relevant questions on a similar three-point scale, indicating the degree of compliance to our trustworthiness requirements. We piloted our survey-based assessment with three use cases of medical AI tools from commercial vendors. This pilot aimed to collect first experiences with this framework to contribute to discussions about potential ways forward to standardize the assessment of transparency and trustworthiness in medical AI. Our assessment pinpointed reporting gaps among all three use cases. Business constraints were major obstacles to providing transparent information about medical AI tools. Our findings motivated a larger study to investigate common pain points for providing transparency for medical AI tools. Secondly, our observations indicated that new strategies may be required to enable an external assessment of transparency while preserving business secrets.

## 2. Materials and Methods

### 2.1. Developing the Survey for Transparent Reporting

Two of our team members with backgrounds in machine learning and epidemiology compiled a semi-open questionnaire to guide transparent model reporting based on the existing guidelines for standard reporting on clinical prediction models and ethical considerations for Appendix A (Table A1). The survey was designed specifically for medical AI use cases that implemented a learning-based machine learning algorithm to predict health outcomes. Three other team members with a background in machine learning, medicine, and business administration reviewed the survey and gave critical input for improvement. The final version of the survey was provided online on the survey platform LamaPoll (https://www.lamapoll.de/ (accessed on 22 August 2022)), because it is compliant with the General Data Protection Regulations (GDPR).

Here, we provide a summary of the survey questions. The full survey can be found in Table A2 in Appendix A. The survey includes 78 questions and is divided into eight sections: The first section includes eight questions to obtain basic information about the developing institution and participant. The next section is the first section for reporting about the use case termed “Section 1” and includes eight questions to report on the intended use of the AI model. Section 2 includes eleven questions to report details about the implemented machine learning (ML) technology. Section 3 includes 24 questions to report about the data that was used to train the model, data preprocessing steps, and data selection for training. Section 4 includes eight questions to report on the legal and ethical considerations during the development. Section 5 includes 13 questions to report technical validation steps that were proposed for medical AI [32] and their respective results on the overall performance, feature importance, comparison to a human expert, fairness and uncertainty, and cost efficiency. Section 6 includes three questions to report about the potential caveats for model deployment.

### 2.2. Participation Procedure

Respondents were recruited through an open call, which was shared via mailing lists and online social networks Linked-In and Twitter, as well as personal contacts from members of our team between 8 May 2021 and 30 September 2021. Respondents stated their interest to participate via e-mail and subsequently received the participation information, consent form, and the link to the survey. Use cases were included in the analysis if the reported model was a medical AI use case using learning-based machine learning. Use cases were excluded from the analysis if the respondent discontinued the survey. Among the selected use cases, our assessor team (experts in machine learning, epidemiology, and business administration) collected their remaining questions about the reported information and clarified those in a follow-up teleconference of 45–60 min with each respondent. Respondents were informed that they can answer clarification questions during the teleconference with ‘Not able to disclose’. If new information was disclosed during the conference, the response was added to the survey-based report. After the teleconference, our team assessed if the report provided the transparency requirements for trustworthy AI. Respondents received exhaustive written feedback, including assessment results and recommendations to improve the transparency and trustworthiness of their AI model.

### 2.3. Transparency and Trustworthiness Assessment

After completing the teleconference session, we assessed transparency across 67 questions within the sections on intended use (Section 1) until caveats and recommendations (Section 6). Table A2 indicates which questions were included for transparency and trustworthiness assessment. Questions (Q) that allowed additional comments on the model development (Q53), validation results (Q75), and caveats (Q78) were considered optional and therefore excluded from the assessment. Two team members (experts in machine learning and epidemiology) scored the responses to each selected question with either 0, 0.5, or 1, indicating if the requested information was not, partially, or fully provided (Table 1). We chose a conservative strategy and scored transparency with 0 if answers were not given, ‘not able to disclose’, ‘unknown’, or ‘no’, e.g., participants gave no consent (Q55), ethical guidelines were not considered during development (Q57), or if the model validation steps were not performed (Section 5). Transparency was scored with 0.5 if partial information was provided. Transparency was scored with 1 point if we rated the provided information sufficiently transparent. Additionally, we assessed whether a set of requirements for trustworthy AI was fulfilled. To this end, we selected 42 questions within the questionnaire, which elicited transparent information recommended by ethical guidelines [11,33]. The selection included questions to specify transparency on the data used for training and validation, questions about participation consent, and potential harm. We defined a set of trustworthiness considerations for a subset of questions (Box 1) The assessors scored these responses with either 0, 0.5, or 1, indicating if the trustworthiness requirement was not, partially, or fully fulfilled. This scoring strategy ensured that the transparency and trustworthiness scores were equal for each question, reflecting that zero transparency also leads to zero trustworthiness. The transparency and trustworthiness scores were calculated as percentages relative to the number of questions that were selected for the assessment. All survey respondents received an exhaustive feedback report including their achieved scores and recommendations to improve compliance with the stated guidelines on reporting and trustworthy AI.

Box 1 **Considerations for the assessment team to score the degree of trustworthiness of the provided answers. The point was assigned if the respective considerations for each question could be answered with ‘yes’.**

Section (1) Intended use of the AI model:

- Q9: Was the intended use specified for a specific clinical task?
- Q10: Is the tool assistive, i.e., designed to include human oversight by a medical expert?
- Q11 and Q12: Is the tool recommended for applications in any setting for the intended use or optimized for specific settings? If applicable anywhere, was the tool sufficiently validated in external validation settings?
- Q15: Was the AI model output specified, and is it appropriate for the intended use?
- Q16: Was the development in close clinical collaboration to ensure medical integrity and safety?

Section (3) Training data information:

- Q29, 34, 36, 38, 39, and 42: Were the training data source, the timeframe of the data collection, the number of samples in the total dataset and subclasses, instruments and settings, and medical image sizes transparently specified?
- Q30: Is the training data accessible for other researchers or regulatory bodies?
- Q43: Was cross-sectional metadata recorded and variables reported? (This information is important to specify requirements for quality assessment)
- Q44: Was missing data reported transparently?
- Q45: Were the inclusion and exclusion criteria reported transparently?
- Q50 and Q51?: Were the training data preprocessing steps, including splitting, reported transparently?

Section (4) Legal and ethical considerations:

- Q54: Was the data anonymized and personal information protected?
- Q55 and Q56: Did individuals give consent that their anonymized data can be used to develop this AI model? If yes, was consent revocable?
- Q57: Were any stated ethical principles considered during product development?
- Q58: Did the model deliberately use sensitive attributes to make predictions?
- Q59: Did the report reflect a performed assessment of fairness (performance stratification among the subgroups)? If yes, which groups were investigated, and was the performance similar across them all?
- Q60: Was potential harm reflected and transparently disclosed?
- Q61: Was the risk of bias across the subgroups mitigated? (Can be scored with one point if the performances across the subgroups were investigated but no differences were found.)

Section (5) Technical validation and quality assessment:

- Q62: Was the model performance assessed on external data?
- Q63, 65: Were the sizes of the total test dataset and classes transparently reported?
- Q64: Were the inclusion and exclusion criteria for the test dataset transparently reported?
- Q66 and Q67: Were the results from the model assessment shared transparently, including performance plots?
- Q68–74: Was the model assessment done across the quality dimensions of bias, fairness, robustness, interpretability, human comparison, and cost efficiency?

Section (6) Caveats and recommendations for deployment:

- Were the caveats for deployment (e.g., regarding underrepresented patient groups or clinical considerations) reflected and transparently reported?
- Were underrepresented groups in data transparently reported and for further performance investigation in those suggested?

## 3. Results

### 3.1. Survey Respondents and Use Cases

Six respondents from different institutions and companies reported about their AI tools using our survey. Three of these tools were excluded as use cases from this analysis for the following reasons: One use case performed a spatiotemporal analysis without a prediction target; therefore, all questions regarding training and validation were not applicable. One use case did not apply learning-based machine learning and instead used precalculated odds ratios as the prediction parameters and therefore could not answer questions on model training. The third use case discontinued the survey, because it was challenging to report all the ensemble tasks from the applied ensemble model.

The remaining three AI tools were included as use cases (UC1–3) in this analysis, as they were clinical prediction models that used leaning-based machine learning. The respondents answered all questions in the questionnaire and completed the questionnaire and subsequent teleconference alone within 45–75 min. The reports cannot be shared to preserve sensitive business information of the participating companies, but we provide a summary of the reported information.

The three respondents had self-reported academic backgrounds in engineering (UC2), computer science (UC1 and UC3), and natural sciences (UC1). The respondents’ experience in machine learning ranged from 1 to 7 years, and all were involved as data scientists in developing the product for 1–4 years. UC1 was developed at a large (>500 employees) company in Germany, UC2 was developed at a small company in India (UC2), and UC3 was developed at a small (<50 employees) company in Germany. UC1 reflected a prediction model to detect one type of cancer in histopathology images. UC2 was a multiclass model to predict the correct placement of endotracheal or gastric tubes using X-rays. UC3 was a model intended for routine care screening that predicts one type of cancer on X-rays. UC1 and -2 were still in the validation phase and not yet available on the market at the time of reporting. UC3 was commercially available on the market. All three use cases applied deep learning-based prediction methods.

### 3.2. Transparency and Trustworthiness Scores

**Figure 1:**
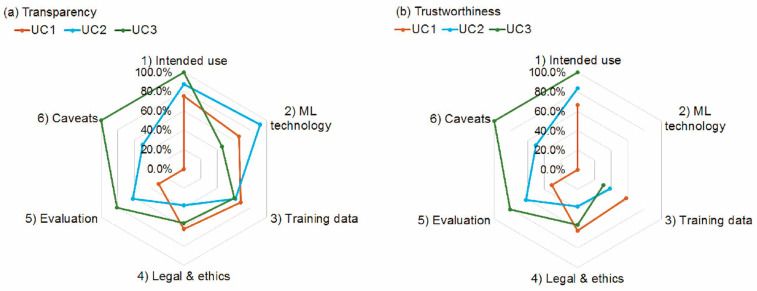
Relative transparency (**a**) and trustworthiness (**b**) scores achieved by use cases (UC) 1–3 in sections (1) Intended use, Section (2) Implemented Machine Learning (ML) technology, Section (3) Training data info, Section (4) Legal and ethical considerations, Section (5) Technical validation and quality, and Section (6) Caveats and recommendations for deployment.

We calculated the absolute and relative transparency and trustworthiness scores for all use cases in total and across all sections of the questionnaire (Table 2 and Figure 1). UC2 and UC3 achieved the highest total transparency scores (both 67.2%), followed by UC1 (59.0%). The total trustworthiness scores were lower than the transparency scores, and the highest was achieved by UC3 (64.3%), followed by UC2 (52.4%) and UC1 (48.8%).

### 3.3. Summary of Assessment Results

We summarized a set of observations that strengthened or reduced the transparency and trustworthiness scores among the three use cases separated by sections in the survey.

#### 3.3.1. Intended Use

Among the eight questions, UC3 achieved the highest level of transparency and trustworthiness (both 100.0%), followed by UC1 (transparency 75.0% and 66.7% trustworthiness) and UC2 (transparency 87.0% and 83.3% trustworthiness). The intended use and clinical considerations of UC2 and UC3 were clearly and transparently specified. We scored 0 points for transparency and trustworthiness for UC2 in Q11 and Q12, because the report disclosed that the tool can be applied anywhere for the intended use without giving enough evidence to support this statement. UC1 could only partially disclose the prediction target due to business constraints.

#### 3.3.2. Implemented Machine Learning Technology

The 12 selected questions in this section were considered for scoring transparency but not trustworthiness. UC2 reached the highest (91.7%) transparency score in this section, because most details on the model development were reported, and the source code could be shared. Details on the implemented machine learning methods and the source code could not be disclosed for UC1 (66.7%) and UC3 (45.8%) due to business constraints.

#### 3.3.3. Training Data Information

UC1 achieved the highest level of transparency (68.8%) and trustworthiness (57.7%), followed by UC2 (transparency: 62.5%, trustworthiness 38.5%) and UC3 (transparency: 60.4%, trustworthiness 30.8%). The training data for UC1 was mixed from a publicly available dataset and data purchased from a data broker. Information on the data collection (geographic location, collector, instruments, and annotation) was only partially available. Information on the timeframe of data collection and laboratory procedures for annotating sample labels were unknown. Instrument types for image acquisition and cross-sectional metadata information could not be disclosed due to business constraints. For UC2, a preprocessed and open-source dataset was used for training. While information about the data provider and data sample sizes were transparently disclosed, details on the data acquisition such as timeframe, instruments for obtaining chest radiographs, annotation, and preprocessing steps were unknown. The dataset used in UC2 did not include cross-sectional metadata variables (demographic or clinical), which we scored with transparency and trustworthiness scores of 0 (Q43). For UC3, details on the acquired dataset (geographic location, timeframe, instruments, annotation, sample size, and missing data) could not be disclosed due to business constraints. UC1 and UC3 reported the potential of domain- and label bias in the training data. Information on splitting and selecting the data for model training was transparently shared by all use cases.

#### 3.3.4. Ethical Considerations

The highest level of transparency and trustworthiness was achieved by UC1 (both 62.5%), followed by UC3 (both 56.3%) and UC2 (both 37.5%). All use cases used deidentified data for developing their AI model (Q54) and no sensitive attributes (i.e., sex, ethnicity, religion, and socioeconomic status) as predictors (Q58), which we scored with 1 point for transparency and trustworthiness. The company of UC1 had not yet assessed the potential of performance differences across different subgroups or harm and did not apply bias mitigation steps, which we scored with 0 points for transparency and trustworthiness (Q59–61). We further scored 0 points for transparency and trustworthiness for UC2 and UC3, because they did not consider any ethical guidelines during model development. The potential harm of UC2 was missed to report in the questionnaire but was transparently disclosed during the teleconference. We counted the response from the teleconference and scored 1 point for transparency and trustworthiness. UC3 reported potential harm in the questionnaire but stated that consent was not necessary as the data was anonymized, which we assigned with zero transparency and trustworthiness points. Performance differences across the subgroups were investigated, but none were found, and the details could not be disclosed, which we scored with 0.5 points for transparency and trustworthiness. For UC2, it was unknown to the company if consent was obtained from the individuals represented in the open-source dataset (Q55 and Q56), which scored 0 points for transparency and trustworthiness. Performance differences across the subgroups were not investigated, because the necessary metadata to form the subgroups were unavailable.

#### 3.3.5. Technical Validation and Quality Assessment

UC3 received the highest level of transparency and trustworthiness (both 80.8%), followed by UC2 (both 61.5%) and UC1 (both 30.8%). All use cases underwent external validation obtained from cohorts that were different from those in the training data. The total sample sizes and selection criteria of the validation data were disclosed for all use cases. For UC3, the overall results were stated (Q66) but plots (Q67) could not be disclosed due to business constraints. Fairness of the predictions (Q68) was assessed across the clinical subgroups but not across the demographic subgroups (0.5 points for transparency and trustworthiness), because the necessary demographic metadata were not available due to data privacy (GDPR) regulations. Steps to assess the model uncertainty and saved costs were reported, but the results could not be disclosed due to business constraints, for which we scored 0.5 points. For UC2, the sample size per label class in the validation data was unknown. The respondent explained in the teleconference that some label classes were missing in the validation data, potentially due to different medical practices between the countries represented in the training and validation data. The performance results were disclosed in a report including plots. The report stated that the model uncertainty was investigated by reviewing false predictions together with a clinician, which we scored with 0 points, because we did not consider this a valid approach. A quality assessment by performance stratification across the subgroups and cost analysis was not yet completed. For UC1, the overall performance results and plots could not be disclosed due to business constraints. The applied methods for a feature analysis were disclosed (Q71); the participant explained that the results were challenging to interpret, because the diagnostic outcome was rare and only a few specialists worldwide are trained to detect the pathology, which we scored with 0.5 points. The rarity of the outcome also challenged the comparison of the AI model performance to a human medical expert, which was not yet conducted. The cost analysis results were only partially reported, because the analysis was not yet completed.

#### 3.3.6. Caveats and Recommendations for Deployment

UC3 scored 100%, UC2 50%, and UC1 0% for transparency and trustworthiness in this section. The report of UC3 reasonably outlined the caveats for deployment application constraints. For UC2, no caveats were initially reported. During the teleconference, the participant outlined that deployment was not recommended to predict one particular outcome class due to low performance during validation, and the tool should not be deployed for children, as the performance was not investigated among this group. The potential boundaries for medical applications were not yet assessed for UC1.

## 4. Discussion

Transparent reporting is a crucial requirement for trustworthy medical AI, but reporting gaps are common despite the many available guidelines. With the motivation to enhance transparency, we developed and piloted a novel survey-based assessment to quantify the degree of fulfilling the transparency and trustworthiness requirements with three medical AI use cases. We discuss our subjective experiences and anecdotal evidence from this assessment.

### 4.1. Survey and Teleconference

The three respondents answered all questions within 45–75 min. The teleconference was useful to clarify answers and explain why certain information was unknown or not able to be disclosed. Reporting caveats for deployment in the survey was difficult for one participant, similar to a previous observation [33,34], but the caveats could be clarified during the teleconference. We acknowledge that our survey may require adaptation to assessors or stakeholders who have different requirements of transparency [33]. New questions may be included in the survey, e.g., to report the primarily intended user groups, dataset update processes [14], ethical approval number [18,24], or registration number and resource of protocols from the validation trials [21]. From the three excluded use cases, we learned that our survey is not suitable for unsupervised spatiotemporal data analysis, ensemble models, and models using predefined parameters. Further applications of our survey to other medical AI use cases are necessary to clarify additional application boundaries, for example, for other algorithms or input data modalities.

### 4.2. Respondents

Our survey requires respondents to have a solid understanding of the development and validation lifecycle of the use case to avoid reductions in transparency scores due to limited knowledge. In our pilot, all three respondents were data scientists who developed the use cases and were able to answer all questions in the survey alone. Answering the survey report alone, however, might introduce subjective reporting errors, recall bias, or reflect the respondents’ perspectives. In our pilot, we were unable to verify if the given responses are true for the use case, and we raised the question of how the truthfulness of the reporting information could be guaranteed.

### 4.3. External Audit

We conducted an external audit using our survey to ensure an unbiased assessment by independent assessors. Currently under debate is which professions should conduct external audits of medical AI tools [31,33]. Similar to another audit frameworks [30], our pilot was conducted by a multidisciplinary team. Our background in epidemiology and medicine helped us to understand the intended use of the product and clinical validation steps. Experience in machine learning was important to identify incorrect technical statements. Knowledge about regulatory and ethical requirements for medical AI tools is important to assess the validity of the answers about legal and ethical considerations, especially when participants report that ‘no consent was necessary’.

One major obstacle for our external audit was business constraints, because they prevented reporting on the intended use, implemented machine learning technology, and used datasets and validation results for two use cases. Similar to a previous report [33], the respondents explained that reporting on the used data threatened their competitive advantage and that the info could only be disclosed for regulatory approval. This observation raises the question if an external audit on the transparency of trustworthiness is only feasible in the post-marketing phase when patents are secured. One use case with business constraints, however, was already available on the market. On the other hand, one use case did not face business constraints to share model implementation details and the source code, suggesting that companies may have varying business constraints. A larger application of our survey is necessary to investigate business constraints in the medical AI sector and how they could be protected to enable external audits of transparency and trustworthiness. It remains to be clarified if external audits should be encouraged in the premarketing phase to ensure compliance with the guidelines before market approval.

### 4.4. Exploratory Results from Use Cases

Our survey-based assessment helped to systematically pinpoint reporting gaps and give specific recommendations to increase compliance with the stated guidelines in a feedback report for participants. We share a set of observed transparency and trustworthiness gaps but would like to note that these cannot be generalized to the general field of medical AI due to the small sample size.

The reporting gaps due to business constraints also reduced the trustworthiness scores, because they prevented disclosing information on the data used for training and validation. Limited insights about the collection process of the externally acquired data were an additional reason for reporting gaps for two use cases. Obtaining informed and revocable consent for using data from individuals is crucial for trustworthy AI [11,12], but we identified this requirement as unfulfilled for two use cases, because the consent procedures were unknown or reported as ‘not necessary’ due to using anonymized data. One company could only perform a limited technical validation because the prediction target was rare, and only a few medical experts could validate the correctness of the predictions. Another company was unable to conduct a fairness assessment across the subgroups, because the demographic metadata was lacking due to data protection, which supports the argument that the potential of medical AI can only be realized if countries specify the right balance between data privacy and data access conditions [34]. Both the transparency and trustworthiness scores from Section 5 on technical validation and Section 6 on caveats for deployment reflected the completeness of the quality assessment lifecycle outlined in [32]. It remains to be verified if the overall scores may reflect the market maturity of the product. Two use cases had the same relative overall transparency score but reported gaps in different sections, suggesting that overall scores may not be comparable between use cases *per se*. The overall trustworthiness score, however, was highest for the use case that was available on the market, because it had completed all the validation steps.

It is not possible to conclude if our provided feedback report with recommendations motivated companies to improve transparent reporting and increase compliance with the stated guidelines, which should be investigated in a future study.

### 4.5. Scoring Transparency and Trustworthiness

Our assessment included a three-point scale (0, 0.5, or 1) to quantify if the reported information fulfilled the transparency and trustworthiness requirements. It is unclear if this simple three-point scale may have biased the calculations of the relative transparency and trustworthiness scores. Future works may consider a 5-point or 10-point scale for a more granular assessment. We acknowledge that the scoring was subjective to our team and may require adaptation to assessors with different expectations. We assumed that each medical AI product has application boundaries [24,31] that should be reported and scored zero transparency and trustworthiness points if these were not disclosed. Similar to a previous study [35], it was challenging to judge if all the potential sources of bias, causes of harm, and caveats for deployment were sufficiently investigated. It was also challenging to judge whether bias mitigation steps are required or not and assign justified scores. Scoring answers on the performed validation steps (e.g., model uncertainty and feature importance) was challenging, because the methods for these validation steps have not yet been standardized and may require adaptation to individual use cases [32,35]. Other assessors may find it relevant to score questions on additional info on model development or validation. Our criteria to assess trustworthiness certainly require adaptation, as they did not include all requirements for trustworthy AI, such as accountability [11,12].

### 4.6. Conclusions and Future Works

We provided a survey-based framework to assess to which degree the transparent reporting and trustworthiness requirements are fulfilled by medical AI products. In our pilot, our quantitative assessment pin-pointed reporting gaps and limitations to fulfill the trustworthiness criteria and helped to give specific recommendations to participants to comply with them. We observed that business constraints and limited information about external data were obstacles to providing transparent information about the three use cases. The next step is to refine our survey by including feedback from a larger group of multidisciplinary stakeholders and administer the survey to a larger sample of companies to investigate if our observations persist in the wider medical AI sector. New strategies may be required to overcome business constraints and enable the disclosure of product information for external audits. It is important to note that our assessment cannot ensure the transparency and trustworthiness of medical AI tools alone. Policies that specify the minimum requirements of transparency for trustworthy medical AI are needed to fulfill the potential of assessments helping to increase the quality of medical AI.

## Figures and Tables

**Table 1:** Scale for scoring transparency and trustworthiness and applied to each question in the questionnaire. Each question was scored with either 0, 0.5, or 1, indicating the degree of transparency of their respective response. The * marks an additional condition for scoring a specific question.

Score	Meaning
0	The answer did not provide information because of any of the following reasons: Missing answer; Non-answer; Not able to disclose; Don’t know; Invalid statement of ‘not applicable’.
0.5	The answer provided partial information. * Q27 and Q28 if the source code and model details were planned to be published.
1	The answer provided sufficient information. * Q61 if the potential of bias was sufficiently investigated.

**Table 2:** Transparency (Trans) and trustworthiness (Trust) scores among all three use cases. Scores are displayed for each section in the questionnaire and among all questions that were selected for the assessment. Transparency and trustworthiness scores are listed as absolute values (x) and percentages relative to the maximum score for each section (%).

	UC1		UC2		UC3	
Section	Trans x (%)	Trust x (%)	Trans x (%)	Trust x (%)	Trans x (%)	Trust x (%)
(1) Intended use	6 (75.0)	4 (66.7)	7 (87.5)	5 (83.3)	8 (100.0)	6 (100.0)
(2) Machine learning technology	8 (66.7)	/	11 (91.7)	/	5.5 (45.8)	/
(3) Training data info	16.5 (68.8)	7.5 (57.7)	15 (62.5)	5 (38.5)	14.5 (60.4)	4 (30.8)
(4) Legal and ethical considerations	5 (62.5)	5 (62.5)	3 (37.5)	3 (37.5)	4.5 (56.3)	4.5 (56.3)
(5) Technical validation and quality	4 (30.8)	4 (30.8)	8 (61.5)	8 (61.5)	10.5 (80.8)	10.5 (84.8)
(6) Caveats and recommendations	0 (0.0)	0 (0.0)	1 (50.0)	1 (50.0)	2 (100.0)	2 (100.0)
**Total**	**39.5 (59.0)**	**20.5 (48.8)**	**45 (67.2)**	**22 (52.4)**	**45 (67.2)**	**27 (64.3)**

## Data Availability

Not applicable.

## Appendix A

**Table A1:** Considerations and guidelines for transparent reporting of clinical prediction models, development of Artificial intelligence (AI), and trustworthy AI. Note that this list is not exhaustive.

	Name of Consideration or Guideline	Author	Focus
1	TRIPOD statement	Moons, K. G. M., Altman, D. G., Reitsma, J. B., Ioannidis, J. P. A., Macaskill, P., et al. Transparent reporting of a multivariable prediction model for individual prognosis or diagnosis (TRIPOD): Explanation and elaboration. *Ann. Intern. Med.* 162, W1–W73, DOI: 10.7326/M14-0698 (2015). Collins, G. S., Reitsma, J. B., Altman, D. G. & Moons, K. G. M. Transparent reporting of a multivariable prediction model for individual prognosis or diagnosis (TRIPOD): The TRIPOD statement. *BMJ* 350, 1–9, DOI: 10.1136/BMJ.g7594 (2015).	Transparent reporting of multivariable prediction models for prognosis or diagnosis
2	Guidelines for developing and reporting machine learning predictive models	Luo, W., Phung, D., Tran, T., Gupta, S., Rana, S., et al. Guidelines for developing and reporting machine learning predictive models in biomedical research: A multidisciplinary view. *J. Med. Internet Res.* 18, 1–10, DOI: 10.2196/jmir.5870 (2016).	
3	Datasheets for Datasets	Gebru, T., Morgenstern, J., Vecchione, B., Vaughan, J. W., Wallach, H., et al. Datasheets for Datasets. 1–28, (2018).	Reporting about datasets that are provided for the development of prediction models
4	Model cards for model reporting	Mitchell, M., Wu, S., Zaldivar, A., Barnes, P., Vasserman, L., et al. Model cards for model reporting. *FAT* 2019—Proc. 2019 Conf. Fairness, Accountability, Transpar.* 220–229, DOI: 10.1145/3287560.3287596 (2019).	Framework to encourage transparent machine learning model reporting
5	Model facts labels	Sendak, M. P., Gao, M., Brajer, N. & Balu, S. Presenting machine learning model information to clinical end users with model facts labels. *npj Digital Medicine* vol. 3 1–4, DOI: 10.1038/s41746-020-0253-3 (2020).	Presenting machine learning model information to clinical end users
6	FactSheets: Increasing trust in AI services through supplier’s declarations of conformity	Arnold, M., Piorkowski, D., Reimer, D., Richards, J., Tsay, J., et al. FactSheets: Increasing trust in AI services through supplier’s declarations of conformity. *IBM J. Res. Dev.* 63, 1–13, DOI: 10.1147/JRD.2019.2942288 (2019).	Multidimensional fact sheets capture and quantify various aspects of the product and its development to make it worthy of consumers’ trust.
7	A roadmap for responsible machine learning for healthcare	Wiens, J., Saria, S., Sendak, M., Ghassemi, M., Liu, V. X., et al. Do no harm: a roadmap for responsible machine learning for health care. *Nat. Med.* 15–18, DOI: 10.1038/s41591-019-0548-6 (2019).	Laying out critical considerations for the development, testing, and deployment of new solutions for a broad audience.
8	ITU/WHO Focus group AI for Health	Wiegand, T., Krishnamurthy, R., Kuglitsch, M., Lee, N., Pujari, S., et al. WHO and ITU establish benchmarking process for artificial intelligence in health. *Lancet* 394, 9–11, DOI: 10.1016/S0140-6736(19)30762-7 (2019).	Standardized audit framework for medical AI
9	CONSORT-AI	Liu, X., Cruz Rivera, S., Moher, D., Calvert, M., Denniston, A. K., et al. CONSORT-AI extension. *Nat. Med.* 26, 1364–1374, DOI: 10.1038/s41591-020-1034-x (2020).	Reporting guidelines for clinical trial reports for interventions involving AI
10	SPIRIT AI	Rivera, S. C., Liu, X., Chan, A.-W., Denniston, A. K. & Calvert, M. J. Guidelines for clinical trial protocols for interventions involving artificial intelligence: the SPIRIT-AI Extension. *Bmj* 370, m3210, DOI: 10.1136/bmj.m3210 (2020).	Guidelines for clinical trial protocols for interventions involving AI
11	STARD 2015 checklist	Bossuyt, P. M., Reitsma, J. B., Bruns, D. E., Gatsonis, C. A., Glasziou, P. P., et al. STARD 2015: An updated list of essential items for reporting diagnostic accuracy studies. *BMJ* 351, 1–9, DOI: 10.1136/bmj.h5527 (2015).	An updated list of essential items for reporting diagnostic accuracy studies
12	DECIDE AI	Vasey, B., Nagendran, M., Campbell, B., Clifton, D. A., Collins, G. S., et al. Consensus statement Reporting guideline for the early-stage clinical evaluation of decision support systems driven by artificial intelligence: DECIDE-AI. *Nat. Med.* 12, 28, DOI: 10.1038/s41591-022-01772-9 (2022).	Reporting guidelines to bridge the development to implementation gap in clinical AI
13	Twenty critical questions on transparency, replicability and effectiveness for machine learning and artificial intelligence research	Vollmer, S., Mateen, B. A., Bohner, G., Király, F. J., Ghani, R., et al. Machine learning and artificial intelligence research for patient benefit: 20 critical questions on transparency, replicability, ethics, and effectiveness. *BMJ* 368, 1–12, DOI: 10.1136/bmj.l6927 (2020).	For developers, editors, patients, clinicians and patients to inform and critically appraise where new findings may deliver patient benefit.
14	Ethics and governance of AI for health	World Health Organization. *Ethics and governance of artificial intelligence for health*. (2021).	Contains key ethical principles for the design and use of AI for health
15	Ethics guidelines for trustworthy AI	High-Level Expert Group on Artificial Intelligence (AI-HLEG), European Commission. *Ethics guidelines for* *trustworthy AI*. *European Commission* https://ec.europa.eu/futurium/en/ai-alliance-consultation.1.html (2019). Available at https://digital-strategy.ec.europa.eu/en/library/ethics-guidelines-trustworthy-ai (accessed on 18 January 2021).	
16	Understanding artificial intelligence ethics and safety	Leslie, D. *Understanding artificial intelligence ethics and safety. The Alan Turing Institute* DOI: https://zenodo.org/record/3240529 (2019) (accessed on 18 November 2021).	A guide for the responsible design and implementation of AI systems in the public sector
17	Evidence standards framework for digital health by National Institute of health and Care Excellence (NICE)	NICE. Evidence Standards Framework for Digital Health Technologies. *Grants Regist. 2019* 540–540, (2019). Available at https://www.nice.org.uk (accessed on 18 November 2021).	Evidence standards framework for digital health
18	Reimagining Global Health through Artificial Intelligence.The Roadmap to AI Maturity by Broadband Commission	Broadband Commission for Sustainable Development, U. Working Group on Digital and AI in Health Reimagining Global Health through Artificial Intelligence: The Roadmap to AI Maturity. (2020).	Actionable recommendations and call to action for advancing countries on their path to AI maturity.
19	Artificial Intelligence/Machine Learning (AI/ML)-Based Software as a Medical Device (SaMD) Action Plan by the U.S. Food and Drug Administration (FDA)	U.S. Food and Drug Administration. *Artificial Intelligence/Machine Learning (AI/ML)-Based Software as a Medical Device (SaMD) Action Plan*. (2021).	Action plan to advance AI/ML-Based Software as Medical Device
20	Eight guiding principles for good Machine Learning practice	U.S. Food and Drug Administration. *Good Machine Learning Practice for Medical Device Development: * *Guiding Principles*. (2021). Available at https://www.fda.gov/media/153486/download (accessed on 18 November 2021).	
21	A Practical Framework for Artificial Intelligence Product Development in Healthcare	Higgins, D., & Madai, V. I. (2020). From Bit to Bedside: A Practical Framework for Artificial Intelligence Product Development in Healthcare. *Advanced Intelligent Systems*, *2*(10), 2000052. https://doi.org/10.1002/aisy.202000052	Product development framework

**Table A2:** Full questionnaire for transparent model reporting. The reporting structured questionnaire contains 79 semi-open questions within the dimensions: (0) Information about the participant, (1) Intended use of the model, (2) Implemented ML technology, (3) Training data information, (4) Legal aspects and Ethical considerations, (5) ML model evaluation and metrics, and (6) Caveats and recommendations. The questions allowed a single answer, unless specified as ‘Multiple answers possible’. The column ‘Suggesting resource’ lists the references for each question that motivated their selection into the questionnaire. The numbers link to references of guidelines and considerations listed in the table above (Table A1). Note that the resources may not be exhaustive. Crosses in the columns ‘Transparency’ and ‘Trustworthiness’ mark whether the question was included for scoring the degree of transparency or trustworthiness.

Questionnaire for Transparent Model Reporting in Artificial Intelligence for Health				
Purpose: This questionnaire elicits details about the development and evaluation process of your AI model implementing machine learning to predict a health outcome. We expect respondents to have a solid understanding of the development and evaluation processes of the use case. Respondents can answer questions in a team or consult colleagues to answer certain questions. We will assess answers with respect to compliance to stated transparent reporting and trustworthy AI guidelines.				
**Question**		**Suggesting Resource**	**Trans-** **parency**	**Trust** **worthiness**
1	What is the name of the use case/project/product/Al model which you will refer to during this questionnaire? □ __________________________	/		
2	Who developed the use case? (e.g., details of developer or team, type of organization (academia, private company, government, etc.) academic institution private company government institution other______________________________	1, 4, 6		
3	In which country was this use case developed? □ _____________________________	/		
4	Are you answering this questionnaire alone or with other team members? alone □ with other team members (please indicate the number and their roles) _____________________________	/		
5	What was your role during the use case development? □ project manager □ product owner □ model developer □ consultant □ data provider □ data scientist □ other: _____________________________	/		
6	What is your academic background? □ computer science or other IT systems □ Engineering □ Medicine □ natural sciences □ social sciences □ economics □ other _________________________	/		
7	For how long have you been working with machine learning? □ less than a year □ 1–2 years □ 3–4 years □ 5–7 years □ 8–10 years □ more than 10 years	/		
8	Please indicate how long you have been involved in the development of the Al model. □ less than a year □ 1–2 years □ 3–4 years □ 5–7 years □ 8–10 years □ more than 10 years	/		
**Section 1—Intended use of the AI model** In the following we will ask questions about the intended application of the model, model output and clinical collaborations.				
9	[Multiple answers possible] Please specify the primary intended use for the Al model Predicting the onset of a health status change_____________ □ Diagnosing a health problem_______________ □ Predicting health risk__________________ □ Surgery planning ________________________ □ Other ______________________	1, 2, 4, 5, 6, 11, 20	x	x
10	Should the Al model work autonomously or assistively? □ Autonomous, to replace health personnel □ Assistive to support health personnel □ Other _____________________________	4, 15	x	x
11	Is your Al model optimized for a specific local or clinical setting (e.g., a specific clinical department, country, etc.)? □ No, can be applied anywhere for the intended use □ Yes, it is optimized for _______________________	1, 2, 4	x	x
12	Could the Al model potentially be utilized for tasks, different from the primary intended use? If yes, please give details. □ No □ Yes, examples are _____________________________ □ Don’t know		x	x
13	[Multiple answers possible] Which of the following clinical considerations apply to your Al model outcome? □ Avoiding ‘False Positives’ is more important than allowing ‘False Negatives’ □ Avoiding ‘False Negatives’ is more important than allowing ‘False Positives’ □ Not applicable □ Don’t know	4	x	
14	[Multiple answers possible] Which form of benefit does your AI allow on the human side? □ gained time □ increased performance compared to the human □ overcoming inter-reader variability potentially increased well-being for humans □ other benefit: ______________	2, 5, 13	x	
15	[Multiple answers possible] Please specify the Al model output. □ Binary classification: The classes are _____________ □ Multiclass (Each sample can only be assigned to one class): The classes are _____________ □ Multilabel (Multiple labels can be assigned to one sample): The labels are ________________ □ Risk score: The scale is__________________ □ Segmented region of interest (ROI). The ROI is... ________________ □ Time until an event occurs. The event is...________________ □ Probability of an event to occur. The event is... ________________ □ Other ____________________	1, 2, 4, 5, 6, 9, 10, 15	x	x
16	Did you consult clinicians during the Al model development? If yes, at which stage? (e.g., Design, data selection, testing) □ No □ Yes, at the stage(s) __________________	6, 7, 15, 20	x	x
**Section 2—Implemented machine learning (ML) technology** In the following we will ask questions about the implemented AI methods for this use case.				
17	[Multiple answers possible] Which ML algorithm was used to build the Al model? □ Decision Tree(s) □ SVM □ Regression □ Autoregressive integrated moving average (ARIMA) □ Convolutional Neural Network □ Generative-Adversarial Network □ Autoencoder □ Recurrent Neural Network □ Bayesian model □ Reinforcement learning □ Other: __________________ □ Not able to disclose	1, 2, 4, 5, 15	x	
18	Was the Al model training supervised, semi-supervised or unsupervised? □ Supervised □ Semi-supervised with ____% labelled data samples □ Unsupervised □ Other _________________________ □ Not able to disclose		x	
19	Please provide more details on the ML method (e.g., for deep learning: architecture with the number of layers and trainable parameters). □ ____________________________________ □ Not able to disclose		x	
20	Does the Al model solve a single task or multiple tasks? (Example for multiple tasks: Segmentation and classification) □ One task □ The following multiple tasks: ____________________________ □ Other: _______________________ □ Don’t know □ Not able to disclose		x	
21	[Multiple answers possible] Which criteria was used to select the best/final Al model during training? (e.g., highest accuracy, F1-score, …)? □ highest accuracy □ highest F1 score □ highest Dice score □ Other: ____________________________________ □ Don’t know □ Not able to disclose	2, 6	x	
22	Does the model make decisions based on predefined thresholds? If yes, please specify those and their clinical significance □ No predefined thresholds used □ Yes the thresholds are: ______________ □ Don’t know □ Not able to disclose	1, 6	x	
23	[Multiple answers possible] Was any technique implemented to speed up the computational process of Al model training? □ No □ Transfer learning from Image Net □ Transfer learning from other pretrained models (please give details on the pretrained model)_________________________ □ Other techniques_________________________ □ Don’t know □ Not able to disclose	6	x	
24	Were any methods applied to reduce overfitting? If yes, please specify hyperparameters. □ No □ Dropout at ______________ □ L1 regularization with _______________ □ L2 regularization with _______________ □ Other methods________________________ □ Don’t know □ Not able to disclose	6	x	
25	Do you have one or multiple selected best Al models? □ Only one best model □ Multiple best models: Please specify the number and the difference between them _________________________ □ Other □ Don’t know	2	x	
26	Please provide any relevant citations of ML methods which were applied. □ Not applicable □ We applied methods based on the following publications ________________________ □ Don’t know □ Not able to disclose		x	
27	Can you share the source code for the model? □ We cannot share the model code because ________________________ □ Yes, it is open source available at ___________________ □ Not yet, but we are planning to make it open-source □ We can share the source code for regulatory approval purposes, if requested □ We can share details about the model, but cannot share the code with anyone □ Other ______________________		x	
28	[Multiple answers possible] Where can we find more detailed information on the Al model? □ The model is reported on arXiv: Link ________________ □ The model is reported in a peer-reviewed journal: Link _____________ □ More information can be found on this website: Link ___________________ □ The model code and documentation is published on Github/Gitlab: Link ______________________ □ The model code and documentation is published elsewhere: Link ______________________________ □ No information published yet, but planning to □ No information published and not planned			
**Section 3—****Training data information** The following questions refer only to the data used during the training stage and not during testing with external data (holdout from training). Questions 29–44 address the original, unprocessed, dataset (raw) and questions 45–53 address the processed data selected for training.				
**(3a) Information about the original unprocessed, unfiltered dataset**				
29	[Multiple answers possible] Please give information where the dataset used to develop the model was collected? Please name known locations □ Countries _______________________ □ Cities ________________________ □ Districts _________________________ □ Hospitals: _________________________ □ Laboratories: __________________________ □ Other: __________________________ □ Don’t know □ Not able to disclose	1, 2, 3, 5, 6, 7, 14, 15, 20	x	x
30	Please report on the availability and accessibility of the data. □ Openly available and free of charge: □ Link ____________________ □ Available on request and free of charge ____________ □ A licence must be purchased at ________________ □ The data was exclusively collected for this project and cannot be shared □ Other ____________________________ □ Don’t know □ Not able to disclose	6	x	x
31	[Multiple answers possible] Who collected the dataset? Please specify the name of the selected option □ A hospital ______________________ □ An academic institution __________________ □ A company ___________________ □ A consortium _____________ □ I don’t know □ Other _____________________________ □ Don’t know □ Not able to disclose	3, 5, 6, 15	x	
32	[Multiple answers possible] Who funded the data collection? Please specify the name of the selected option □ A company ___________________ □ A funding institution ______________________ □ Other _________________ □ Don’t know □ Not able to disclose	1, 3, 6, 9, 10, 11	x	
33	What was the purpose of collecting the data? □ ______________________________________________ □ Don’t know □ Not able to disclose	3, 6, 7, 15	x	
34	When was the data collected? Please specify the timeframe. □ ____________________________________________ □ Don’t know □ Not able to disclose	1, 2, 3, 5, 6, 7, 9, 10, 14, 15, 20	x	x
35	What does one data sample represent? Please specify. □ One individual □ One time point □ One image patch □ One other ___________________________ □ Not able to disclose	3, 6	x	
36	How many total data samples does the original dataset contain? □ less than 100 □ 100–599 □ 600–999 □ 1000–5999 □ 6000–9999 □ 10,000–99,999 □ 100,000–499,999 □ 500,000–1 Mio □ More than 1 Mio □ Don’t know □ Not able to disclose	1, 2, 3, 5, 6	x	x
37	[Multiple answers possible] Which data modalities are included in the original dataset? Please specify. □ CT/MRI/X-ray/PET images: _________________ □ Medical text reports □ Laboratory test results: ___________________ □ Cognitive test results: ___________________ □ Genetic data: _____________________ □ Microscopy images ____________________ □ Other __________________________ □ Don’t know □ Not able to disclose	3, 6	x	
38	[Multiple answers possible] Which instruments and settings were used to capture the data? □ Camera type and settings ___________________ □ Microscope type and settings ____________________ □ Laboratory assays and tests _______________________ □ Other _______________________ □ Don’t know □ Not able to disclose	3, 4, 6	x	x
39	If the dataset contained images: Please specify the image size of the original (raw) images. □ Not applicable □ All raw images had the image size: _____________________ □ All raw images had varying image sizes in the range of _____________________ □ Information of raw images is not available. The image size of processed available images was _______________ □ Don’t know □ Not able to disclose	3, 6	x	x
40	Are individuals represented at one or at multiple timepoints in the original dataset? If multiple, please specify time intervals and irregularities. □ All individuals are represented only at one timepoint □ Some individuals were recorded only at one timepoint, some at multiple timepoints, depending on ___________________ □ All individuals were recorded at multiple timepoints in regular intervals □ All individuals were recorded at multiple timepoints in various intervals depending on _________________ □ Other _______________________ □ Don’t know □ Not able to disclose	3, 6	x	x
41	Are data samples annotated with labels? If yes, how and by whom were these annotated? □ No label associated with data samples □ Yes, the labels were annotated by an algorithm □ Yes, the labels were annotated by X (number) human experts with X (number) years of experience □ Yes, the labels were obtained from a laboratory test result ____________ □ Yes, the labels were obtained by ___________________ □ Other ____________________ □ Don’t know □ Not able to disclose	1, 2, 3, 6, 14, 20	x	x
42	[Multiple answers possible] How many samples of each label class were present in the original dataset? □ Not applicable □ Class 1: (class name, % of samples relative to total) ___________ □ Class 2: (class name, % of samples relative to total) □ Class 3: (class name, % of samples relative to total) □ Class 4: (class name, % of samples relative to total) □ Class 5: (class name, % of samples relative to total) □ More classes: _____________________________ □ Don’t know □ Not able to disclose	1, 2, 3, 6, 9	x	x
43	[Multiple answers possible] Does the dataset record cross-sectional metadata? Please select present variables and specify the frequencies or appropriate summary statistics. □ age: _______________________ □ sex: _______________________ □ ethnicity: ______________________ □ religion: ________________________ □ type of healthcare visit (routine/emergency): __________________________ □ stage of disease □ severity of disease □ time after first diagnosis □ time after onset of symptoms □ time after hospital admission □ symptoms: ________________________ □ comorbidities: ___________________________ □ Treatment, past or current: _________________ □ Other variables: ___________________________________ □ Don’t know □ Not able to disclose	1, 2, 3, 4, 6	x	x
44	[Multiple answers possible] Did you encounter any missing data in the original dataset? If yes, please specify affected variables or data-modalities, missing fraction relative to all entries and potential reasons for missing data. □ All data entries were complete □ The following variables/data modalities were missing. (Missing fractions in %) ________________________ □ Data was missing for unknown reasons □ Data was missing if/because ______________ □ Other _____________________ □ Don’t know □ Not able to disclose	1, 2, 3, 6, 14	x	x
(**3b) Information about data selection and preprocessing** to prepare data for model development, comprising training and validation. This excludes testing on hold-out data.				
45	[Multiple answers possible] How many samples/individuals were selected from the original dataset for developing the model? □ All samples/individuals from the original dataset were selected for developing the model □ A subset was selected for model development. Selection criteria and fraction relative to the original dataset were ______________________________ □ Instances/individuals were excluded from model development, if/because _______________________ □ Don’t know □ Not able to disclose	1, 2, 3, 5, 6, 14, 15	x	x
46	Did you encounter any errors, sources of noise, redundancies present in the original dataset which were relevant for selecting the data for training? If yes, please provide a description and how you handled them. □ None □ Yes, __________________________ □ Don’t know □ Not able to disclose	2, 3, 6, 14	x	
47	[Multiple answers possible] Which data modalities or variables were selected for the processed dataset as model input? Please choose relevant categories and specify within. □ CT/MRI/X-ray/PET/ images: _________________ □ Microscopy images: ____________________ □ Medical text reports: _____________________ □ Laboratory test results: ___________________ □ Genetic data: ____________________ □ Cognitive test results: ___________________ □ Other __________________________ □ Not able to disclose	1, 2, 3, 5, 6, 14	x	
48	[Multiple answers possible] Which preprocessing steps were performed to prepare data for ML model development? □ Resizing/compressing/cropping images to _______________________ □ SIFT feature extraction ____________________ □ Text processing _______________________ □ Missing data imputation using ______________ □ Normalization of image pixel values ___________________ □ Normalization of numeric variables ___________________ □ Other ________________________ □ Don’t know □ Not able to disclose	1, 2, 3, 6, 14	x	x
49	[Multiple answers possible] By which proportions did you split the preprocessed data samples into training, validation and test set? □ Training _____________% □ Validation ___________% □ Test (holdout from model development)_______________% □ Don’t know □ Not able to disclose	2, 6, 7	x	
50	Did you assign samples to each split at random or stratified by any criteria? □ At random □ By matched case-control ___________________ □ Stratification criteria _____________________ □ Don’t know □ Not able to disclose	2, 6, 7	x	x
51	Did you apply k-fold cross validation? □ No □ Yes, stratified at random into fold k = _________ □ Yes, stratified by the following variables into folds k = ___________________ □ Don’t know □ Not able to disclose	2	x	
52	If k-fold cross validation was applied, was the test set held separate or was it mixed with the validation folds? □ Not applicable □ We did not separate a test set from k-fold cross validation □ We separated a test set from k-fold cross-validation □ Don’t know □ Not able to disclose	2	x	
53	Any other comments or relevant information about model development, which was not addressed previously? □ No □ Yes, _____________________________			
**Section 4—Ethical considerations**				
54	Were the datasets de-identified or anonymised so that individuals cannot be identified? □ No □ Yes □ Other _________________________	3, 14, 15	x	x
55	Did individuals who are represented in this data give consent for using their information developing this use case? □ Consent was not necessary □ Oral consent was obtained □ Written consent was obtained □ Other ______________________ □ Don’t know	3, 6, 10, 14, 15	x	x
56	Were individuals provided with any mechanism to revoke their consent in the future or for specific uses? □ Not applicable □ Consent can be revoked □ Consent cannot be revoked □ Other___________________ □ Don’t know	3, 6, 10, 14, 15	x	x
57	Which kind of ethical considerations did you follow in your product development (e.g., from EMA, FDA, WHO,…)? □ We did not follow any particular ethical considerations □ Yes, we followed ethical considerations stated by ______________ □ Other________________________ □ Don’t know □ Not applicable	/	x	x
58	[Multiple answers possible] Does the Al model use any sensitive attributes to make predictions? If yes, please specify the attributes. □ No □ Ethnicity □ Sex □ Religion □ Age □ Other ___________________________ □ Don’t know □ Not able to disclose	4, 15	x	x
59	Are there any subgroups in which the model might have lower or higher performance compared to others? □ We do not anticipate that there are subgroups with lower/higher performances, because ______________________________ □ Possibly, but we have not investigated this (yet). □ We anticipate different performances within the following subgroups _________________________________ □ but we haven’t investigated this in detail yet. □ We found performance differences within the following subgroups _________________________________ □ Other_________________________ □ Not able to disclose □ Don’t know	6, 7, 9, 13, 14, 15	x	x
60	[Multiple answers possible] What are potential harms if model predictions are false?Please try to estimate the (1) likelihood that this harm occurs in an application setting and the severity of harm and give reasons for your rating. □ Not applicable □ The likelihood is estimated to be low (0–10%) □ The likelihood is estimated to be medium (11–40%) □ The likelihood is estimated to be high (41–100%) □ The severity of potential harm is estimated to be low because _________________________ □ The severity of potential harm is estimated to be medium because _________________________ □ The severity of potential harm is estimated to be high because _________________________ □ Other __________________________ □ Don’t know □ Not able to disclose	4, 9, 14, 15	x	x
61	Did you apply any mitigation strategies to overcome risk of bias across sensitive attributes? If yes, please specify the method and results. □ Not applicable, because ______________________________ □ Not yet □ Yes, we applied _____________________________________ □ Don’t know □ Not able to disclose	4, 6, 7, 14, 15	x	x
**Section 5—Technical validation and quality assessment**				
62	Which type of evaluation will you report in the following? (Please choose the type with the highest relevance for regulatory approval) □ Performance on the training set □ Performance on validation data, which has also been used during training □ Performance on test data, which has been excluded from training, but was split from the original dataset □ Performance on test data, which has been excluded from training, and obtained from an external source which is different from the training data (not a split). Please specify how, when and where this evaluation data was collected, and how it’s demographics differ from the training data ________________________ □ Not able to disclose	2, 6, 9,10, 11, 14, 15	x	x
63	How many total data samples does the evaluation dataset contain? □ less than 100 □ 100–599 □ 600–999 □ 1000–5999 □ 6000–9999 □ 10,000–99,999 □ 100,000–499,999 □ 500,000–1 Mio □ More than 1 Mio □ Not able to disclose □ Don’t know	1, 2, 3, 6, 9, 11, 14	x	x
64	[Multiple answers possible] Please specify the inclusion and exclusion criteria for samples/individuals in the test dataset. □ All available data for testing was included in the test set □ The selection was at random □ The selection was based on the following criteria ___________________________________ □ Samples were excluded from testing if _____________________ □ Don’t know □ Not able to disclose	1, 2, 3, 4, 6, 9, 10, 11, 14, 15, 20	x	x
65	[Multiple answers possible] How many samples of each label class were present in the test dataset? □ Not applicable □ Class 1: (Name, percent relative to all test samples) ___________ □ Class 2: (Name, percent relative to all test samples) ___________ □ Class 3: (Name, percent relative to all test samples) ___________ □ Class 4: (Name, percent relative to all test samples) ___________ □ Class 5: (Name, percent relative to all test samples) ___________ □ more classes (Name, percent relative to all test samples) __________________________ □ Not applicable □ Don’t know □ Not able to disclose	1, 2, 5, 11, 14	x	x
66	[Multiple answers possible] Which performance measures are reported for this evaluation? Please specify the gold standard and respective results. □ Accuracy: ________________________________ □ F1-Score: ________________________________ □ Sensitivity: ________________________________ □ Specificity: ________________________________ □ Precision: ________________________________ □ Recall: ________________________________ □ Dice score: ________________________________ □ Area under the curve: ________________________________ □ Area under the precision-recall curve: ________________________________ □ Calibration: ________________________________ □ Other: ________________________________ □ Not able to disclose □ Don’t know	1, 2, 4, 5, 6, 7, 9, 10, 11, 14, 15, 20	x	x
67	Can you provide relevant plots and tables about the evaluation results (e.g., ROC-AUC plot) □ No, because __________________________________ □ Yes, we can share the plots upon your request	/	x	x
68	[Multiple answers possible] Did you investigate Al model performance variations across different groups? If yes, please specify the groups and report the results here. □ No □ Age groups: _______________________ □ Sex: _______________________ □ Ethnicity: ______________________ □ Type of healthcare visit (e.g., routine/emergency): __________________________ □ Stage/severity/time after onset of disease: _______________ □ Symptom groups: ___________________________ □ Comorbidity groups: ___________________________ □ Other features ___________________________________ □ Don’t know □ Not able to disclose	4, 6, 7, 13, 14, 15, 20	x	x
69	[Multiple answers possible] Are there output classes or groups (see previous question) for which the Al model performed worse compared to others? □ Not applicable □ The performance was similar for all classes and groups □ We found performance differences within the following classes/groups __________________________ □ Don’t know □ Not able to disclose	4, 6, 11, 13, 14, 15, 20	x	x
70	Have you applied statistical testing to compare Al model performance across different groups? If yes, specify the tests and significance level of *p*-values applied. □ Not applicable □ No □ Yes, the following test(s) and significance level: _____________________ □ Don’t know □ Not able to disclose	4, 6, 9, 13	x	x
71	Did you perform an analysis to determine which features were most important to predict the model output? E.g., SHAP, class-activation or saliency maps? If yes, how was it done and which input features were most important? □ Not applicable □ No □ Not yet, but planning to □ Yes, applied method and results were: _______________________________________. □ Don’t know □ Not able to disclose	6, 7, 14, 15	x	x
72	Did you use approaches to assess uncertainty and variability in model output? If yes, which methods and what were the results? □ Not applicable □ No □ Not yet, but planning to □ Yes, the analysis approach and results were ___________________________________________ □ Not able to disclose	4, 6, 7, 15	x	x
73	Did you compare the model performance to one or more human experts? If yes, describe the analysis approach, competence level of the human, gold standard and results (e.g., conditions, under which the machine or the human performs better) □ Not applicable □ No □ Not yet, but planning to □ Yes, the analysis approach and results were ___________________________________________ □ Not able to disclose	20	x	x
74	Did you perform a cost-efficiency (e.g., saved human hours) analysis to quantify to which extent the application of your model can save healthcare costs? If yes, describe the analysis approach and results. □ Not applicable □ No □ Not yet, but planning to □ Yes ___________________________________________ □ Not able to disclose	2, 7, 13	x	x
75	Any other evaluation results which you would like to report? Here is space for additional information. □ No □ Yes _______________________			
**Section 7—Caveats and recommendations for deployment** Are there any caveats or recommendations for applying the product correctly or safely?				
76	[Multiple answers possible] Are there relevant subgroups that were not represented or under-represented in the validation dataset and in which Al model performance should be investigated? □ Not applicable □ No, all relevant subgroups were represented in the data and further investigation is not necessary □ The following subgroups were not/or under-represented in the evaluation data and we recommend further testing for these ________________________________ □ Don’t know □ Not able to disclose	1, 2, 4, 5, 6, 7, 9, 14, 15, 20	x	x
77	Are there medical contexts or populations in which the reported use case is not recommended / advisable to be applied? □ No □ Yes, _____________________________________ □ Don’t know □ Not able to disclose	1, 2, 4, 5, 7, 9, 14, 15, 20	x	x
78	Are there additional recommendations or caveats for deploying the product? □ No, everything stated previously □ Yes, ______________________________________ □ Don’t know □ Not able to disclose	1, 4, 5, 14, 15, 20		

## Appendix B

### Appendix B.1. Participant Information and Consent

Hello,

Thank you very much for your interest in participating in our survey on transparent model reporting for trustworthy machine learning for health!

This survey is conducted by ANONYMIZED FOR REVIEW to establish a standardized assessment framework for the evaluation of AI-based methods for health, diagnosis, triage, or treatment decisions.

Purpose of the processing

Guidelines to establish medical AI approval frameworks are currently under development, and transparent model reporting has been suggested as an important requirement to build trustworthy medical AI. It is currently unclear if current practices fulfil the reporting requirements, especially when the algorithm is proprietary or trained with protected data; it might be possible that not all information can be disclosed. The goal of this study is to assess the level of transparency and trustworthiness of medical AI tools. We invite participants who were involved in developing medical AI tools to provide information about their use case at the highest transparency level as possible. We will guide transparent reporting by our questionnaire compiled from previous considerations. We will investigate the current practices of transparent model reporting for medical AI and pinpoint challenges. With our findings, we aim to help (1) product owners to adapt to regulatory requirements and (2) regulatory institutions to assess the feasibility of fulfilling the stated reporting requirements.

Participation procedure:

This survey will ask detailed information about the development of your use case spanning the following domains:

- (0) Information about the participant;
- (1) Intended use of the medical AI product;
- (2) Implemented machine learning (ML) technology;
- (3) Training data information;
- (4) Ethical considerations;
- (5) Technical validation and quality assessment;
- (6) Caveats and recommendations for deployment.

The questions are semi-open and provide multiple-choice answer options but also leave space for individual answers. If certain information cannot be disclosed, participants may choose the answer option ‘Cannot be disclosed’. Other answer options include ‘Don’t know’ and ‘Not applicable’. We will not ask you about personal data of patients.

We expect that filling out the survey will require 45–60 min. Please submit the questionnaire within 3 weeks.

We will investigate the transparency level of your survey response qualitatively. The analysis will be carried out only by participating researchers from the ANONYMIZED FOR REVIEW group. If you wish, we will provide you a short feedback report summarizing our conclusions from your survey. After we provide you feedback on the assessed transparency, we will ask for your feedback about model reporting in a small follow-up survey. In this feedback, you can tell us openly if the survey was helpful to provide for relevant questions or if and why it was difficult to provide the enquired information.

Publishing results

We plan to publish the results from this survey, which entails a summary of the current practices of transparent model reporting and challenges. We will anonymize your name, the name of your use case, and the name of the institution which developed the model. Your provided information on methodological details of the model will not be published. We will only publish the level of transparency and trustworthiness you provided in your report. You and your employing institution can choose to be mentioned in acknowledgements or remain anonymous. We will ask you if you want to be mentioned in acknowledgements separately after you completed the survey.

Privacy Policy

#### Appendix B.1.1. Contact

The responsible body within the meaning of the General Data Protection Regulation (GDPR) is: ANONYMIZED FOR REVIEW.

As the responsible body, we implement all legally required measures to protect your personal data. If you have any questions about this data protection declaration or about the processing of your personal data, please contact our company data protection officer: ANONYMIZED FOR REVIEW.

#### Appendix B.1.2. What Does the Privacy Policy Apply to?

This data protection declaration always applies when we process your personal data (i.e., collect, save, use, transmit, or delete your personal data).

#### Appendix B.1.3. What Personal Data Do We Collect from You?

ANONYMIZED FOR REVIEW collects and processes your contact information, such as first and last name, business e-mail address, your job position, your employer, and your academic background. (We ask for your e-mail address if you wish to receive feedback on the transparency level and participate. The e-mail address can be given voluntarily.)

#### Appendix B.1.4. Legal Basis

Unless expressly stated otherwise, the legal basis for data processing is your expressly granted consent in accordance with Article 6, paragraph 1, sentence 1 lit. (a) GDPR\.

#### Appendix B.1.5. Who Will Get Your Data?

The survey is conducted by ANONYMIZED FOR REVIEW. The data from this project will be processed by ANONYMIZED FOR REVIEW. We do not forward your personal information to any persons other than the FGAI4H members stated above or to any third parties!

### Appendix B.2. What Are Your Rights?

You are granted various rights when it comes to the processing of your personal data based on Articles 15–21 GDPR. To exercise your rights, please write us an e-mail ANONYMIZED FOR REVIEW or contact our data protection officer mentioned above.

#### Appendix B.2.1. Your Right to Withdraw

You are entitled to withdraw this consent at any time with effect for the future. The processing of personal data will remain lawful until the date of receipt of your cancellation notice.

#### Appendix B.2.2. Your Right to Information and Correction

You can request information about your personal data that we have processed. Should your data no longer be valid or applicable, you can request a correction. If your data should be incomplete, you can request its completion. If we have passed on your data to third parties, we will inform these third parties about the correction, insofar as this is required by law.

#### Appendix B.2.3. Your Right to Deletion of Your Personal Data

You are entitled to request the deletion of your personal data if:

○ your personal data is no longer required for the purposes for which it was collected,
○ you have withdrawn your consent and there is no other legal basis,
○ you object to the processing and there are no overriding legitimate grounds to justify processing,
○ your personal data has been processed unlawfully, or
○ your personal data must be deleted in order to comply with the legal requirements.

#### Appendix B.2.4. Your Right to Restrict the Processing of Your Personal Data

You have the right to request that the processing of your personal data be restricted if:

○ the accuracy of your personal data is contested by you until we can prove the accuracy of the data,
○ the processing is not lawful;
○ your data is no longer required for the purposes of processing but you need it to assert, exercise, or
○ defend yourself against legal claims; or
○ you have raised an objection, as long as it is not yet been determined whether your interests prevail.

#### Appendix B.2.5. Your Right to Object

We may process your data on the basis of legitimate interests or in the public interest. In these cases, you have the right to object to the processing of your data. In the event of an objection, we will then only continue processing your personal data if the compelling legitimate reasons for the processing of this data demonstrably outweigh your interest in non-processing.

#### Appendix B.2.6. Your Complaint Right

If you are dissatisfied with our response to your request in individual cases, you are entitled to lodge a complaint with the data protection officer and the responsible supervisory authority. The responsible supervisory authority is the ANONYMIZED FOR REVIEW.

#### Appendix B.2.7. Your Right to Data Transferability

You have the right to receive your personal data from us in a transferable and conventional format.

#### Appendix B.2.8. How Long Do We Store Your Data?

All personal data will only be stored for as long as is necessary for the stated purpose. It will be deleted at the end of the follow-up survey. If a participant did not proceed to the follow-up survey phase, the data will be deleted at the end of the survey phase for all other participants.

### Appendix B.3. Consent and Link to Survey

If you have read and understood all the information provided above, you may proceed to the survey. By submitting your survey response at the above provided link, you consent to participation, storing, and processing your data and information for this study purpose.
